# He Wants to Know

**Published:** 1921-12

**Authors:** N. Parker


*To the Editor of* THE HOSPITAL AND HEALTH REVIEW.

### He Wants to Know.

*To the Editor of* THE HOSPITAL AND HEALTH REVIEW.

SIR,—I wonder whether any of your readers can answer the following questions:

Has the Society of Medical Officers of Health taken any steps to bring to the notice of the Corporations of Manchester and Liverpool the salaries which should be offered for the positions shortly to become vacant?

What will happen to County Nursing Associations when their probationers realise that a half-trained nurse will not be able to get on to the Nursing Register after 1925?—I am, Sir, yours truly,

N. PARKER.

---

### "NOTABLE EPIDEMICS."

We regret that, owing to other calls upon our space, we are unable to print this month the third of a series of articles on "Notable Epidemics," by Dr. A. T. Nankivell. We hope, however, to find room for his article on "The Great Plague" in our January number.—EDITOR.

